# Totally Laparoscopic Resection of an Extremely Giant Hepatic Hemangioma

**DOI:** 10.1055/s-0039-1698520

**Published:** 2019-09-21

**Authors:** Fadl H. Veerankutty, Shiraz Ahmad Rather, Varghese Yeldho, Bincy M. Zacharia, Shabeer Ali TU, Venugopal B.

**Affiliations:** 1 Department of Hepatobiliary Pancreatic and Liver Transplant Surgery, Kerala Institute of Medical Sciences, Trivandrum, Kerala, India

**Keywords:** liver hemangioma, laparoscopic liver resection, laparoscopic anterior approach, preoperative embolization, anatomical liver resection, pure laparoscopic hepatectomy

## Abstract

Risk of massive intraoperative hemorrhage and the difficulty to control it makes the laparoscopic treatment of giant hepatic hemangiomas (GH) a challenge for minimally invasive hepatobiliary surgeons. Symptomatic GHs of more than 20 cm (extremely giant hepatic hemangiomas) are typically treated with an open resection. There is a paucity of literature on laparoscopic resection of extremely giant hepatic hemangiomas. We describe (with video), here, the technical nuances of pure laparoscopic resection of an extremely giant hepatic hemangioma using modified port positions and the anterior approach.

Laparoscopic resection of liver lesions has been associated with a diminished stress response resulting in an early recovery and a shorter hospital stay along with an improved cosmetic outcome compared with open liver resection. Because of technical difficulties and the possibility of catastrophic intraoperative bleeding, the role of laparoscopy in the management of symptomatic giant hepatic hemangioma (GH) had been restricted based on site and size criteria. 1 The rapid progress made in the field of laparoscopic surgery in recent years has proffered great opportunities for resecting symptomatic liver hemangiomas in a minimally invasive fashion. Liver hemangioma of more than 20 cm in size is generally termed as an extremely giant hepatic hemangioma. 2 The literature on laparoscopic resection of extremely GH is scarce as these are typically resected using the open approach. 2 3 4 Here, we describe the technical nuances of totally laparoscopic removal of an extremely giant hepatic hemangioma of 22-cm size in craniocaudal diameter and occupying almost the entire right lobe using modified port positions and the anterior approach.

## Patient Details

**Fig. 1:**
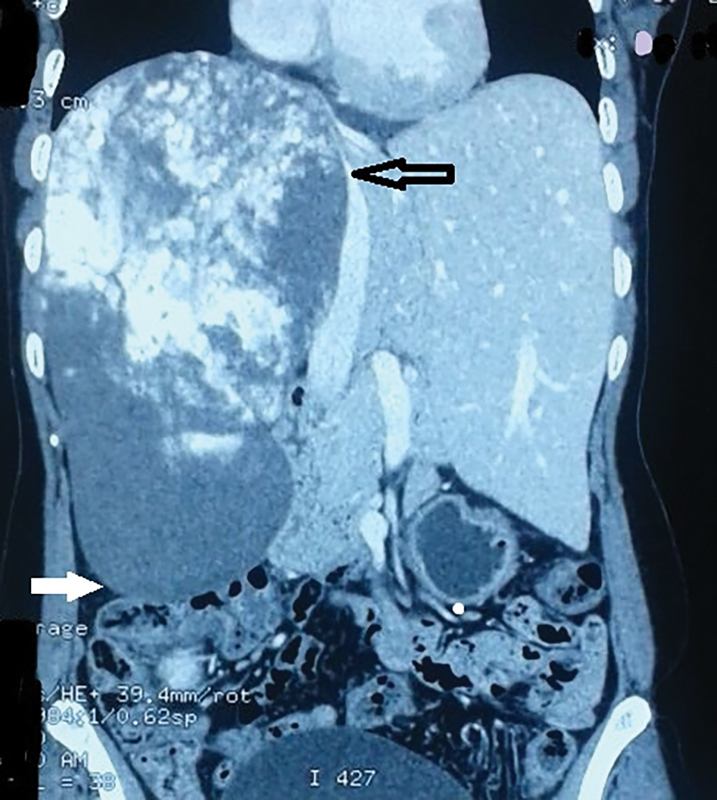
Contrast enhanced CT scan demonstrates the huge size of hemangioma which occupies almost the entire right lobe. CT, computed tomography. (Open black arrow shows the medial extend and the solid white arrow shows the inferior extent of the hemangioma).

A 51-year-old female was referred to our institute with a history of progressive right upper quadrant pain of 5 months duration. Her routine blood tests were essentially normal. Imaging of the abdomen revealed a hemangioma of 22 cm in craniocaudal diameter involving almost the entire right lobe and extending up to the right iliac fossa ( Fig. 1 ). In view of persistent symptom, the patient was planned for laparoscopic right hepatectomy after obtaining informed consent and thorough preoperative work up. Preoperative transarterial embolization of GH was performed but it could not reduce the size of the GH, possibly due to the presence of collateral vessels. 3

## Procedure

### Port Placement

The patient was placed in the supine low-lithotomy position. The surgeon was standing in between the patient's legs, with two assistants, one on each side of the patient. Because of the huge size of the GH, routine supraumbilical camera port and bilateral upper quadrant working ports for standard right hepatectomy could not be placed. Hence the pneumoperitoneum was created using a suprapubic 10-mm camera port inserted by open method. Two 5-mm working ports, one in each iliac fossa were inserted for inflow control which also helped in performing initial stages of the parenchymal transection. After obtaining inflow control and performing a major part of the parenchymal transection, the camera port was shifted to the umbilicus for outflow control and mobilization of the liver. An additional 10-mm port was placed below the left-costal margin slightly lateral to the midclavicular line for parenchymal transection and right hepatic vein (RHV) stapling. Later on, a 5-mm working port was placed in the midclavicular line in the right upper quadrant ( Video 1 ; available online only).

**Video 1**

Demonstration of patient details and port placement.

### Inflow Control

The falciform ligament was taken down, following which the suprahepatic vena cava was also dissected. Access to the right pedicle was gained after dividing the cystic duct and artery and lowering the hilar plate. The gall bladder was kept undissected and it was used for traction when needed. At this stage, a few collateral vessels in the perihilar region were clipped and divided. Following this, the right hepatic artery (RHA) and the right portal vein (RPV) were dissected and looped. A caudate branch from RPV was divided. The caudate lobe was freed from the inferior vena cava (IVC). Only RPV was needed to be clipped to develop the ischemic line of demarcation since RHA had already been embolized ( Video 2 ).

**Video 2**

Demonstration of steps of inflow control.

### Parenchymal Transection and Outflow Control:

We decided to do a right hepatectomy as the right lobe was almost entirely replaced by the GH. The anterior approach was preferred as the huge size of the hemangioma hampered early mobilization of the right lobe. Initial superficial transection was done by the harmonic scalpel and in deeper planes, the ultrasonic aspirator (CUSA) was used. The middle hepatic vein (MHV) was safe guarded and used as a guide for liver transection plane. Draining veins from segment V and VIII were clipped or stapled depending on the size. After partial parenchymal transection, the attention was redirected towards the inflow vessels. Hem-o-lok clips were applied on RHA and RPV and they were divided with an Endo GIA vascular stapler. The right hepatic duct along with its accompanying hilar plate was divided with an Endo GIA vascular stapler. The parenchymal transection was proceeded cephalad and deeper planes were developed carefully safeguarding IVC. After completion of hepatic parenchymal transection, the outflow control was achieved by exposing RHV and transecting it by an Endo GIA vascular stapler ( Video 3 ).

**Video 3**

Demonstration of parenchymal transection, outflow control, and specimen delivery.

### Mobilization of the Right Liver

This part of the surgery was time consuming due to engorged GH. Few small retroperitoneal collateral vessels were clipped and divided by minimal lifting of the GH from the diaphragm. The right lobe containing GH was mobilized meticulously from the retroperitoneum and the specimen was completely freed from the IVC. Special attention was paid to attain complete hemostasis. Finally, the contents of the hemangioma were aspirated and the specimen was retrieved in a bag through a pfannenstiel incision. The left lobe was then fixed to the remnant falciform ligament to prevent the spontaneous rotation into the right subphrenic space. A right flank drain was kept in the surgical field which was removed on the 1st postoperative day ( Video 3 ).

The operative duration was 320 minutes and the blood loss was 200 mL. Patient had an uneventful recovery and was discharged on the 4th postoperative day.

## Discussion

Hepatic hemangioma can generally be observed and requires intervention only if it is symptomatic. Hepatic hemangiomas of more than 5 to 10 cm are called giant hepatic hemangiomas. Symptomatic GH is traditionally treated surgically either by enucleation or resection depending on the location and size of the tumor. Enucleation is favored over resection, if feasible. We adopted the right hepatectomy because the hemangioma had replaced almost the entire right lobe.

The Louisville Statement, 2008, considered hemangioma of more than 5 cm as a relative contraindication for laparoscopic resection. 1 With the advent of better sophisticated devices, like laparoscopic harmonic/CUSA probes and Endo GIA staplers, complex laparoscopic liver resections can be safely performed by experienced surgeons. Kim and Kwon retrospectively evaluated the feasibility of laparoscopic removal of hepatic GH greater than 6 cm in diameter (6–18 cm) in nine patients. 5 All, except one of nine, patients underwent anatomical resection. There was no major morbidity or mortality in their series. It was followed by the description of a handful of different techniques for the resection of GH, mostly between 10 and 18 cm. Lanthaler et al reported laparoscopic resection of a 20-cm pedunculated hemangioma after reducing its size by preoperative embolization. 4 Preoperative embolization may not always reduce the size of the hemangioma. In a series of GH more than 20-cm operated by open technique, the mean size of the tumor did not show any significant change on follow-up imaging studies after preoperative embolization. 3 Zhang et al showed that a combination of infrahepatic IVC clamping and the Pringle's maneuver with laparoscopic extracapsular enucleation of GH can significantly reduce the intraoperative blood loss and transfusion rates compared with the Pringle's maneuver alone. 6 But these vascular exclusion procedures are not always needed during laparoscopic resection of GH. 4

Our case is one of the largest GHs reported to be resected laparoscopically. 4 5 We did not use the Pringle's maneuver or IVC clamping. We planned the anterior approach, as an early forceful retraction during mobilization of the GH in the conventional approach might result in serious complications like rupture of GH leading to massive hemorrhage and even exsanguination of the patient. In the anterior approach, after the inflow control, the parenchymal transection is performed from the anterior surface of the liver until the anterior surface of the IVC is exposed and then all venous tributaries, including RHV, are controlled before the mobilization of the right lobe of liver and the tumor. 7 Besides the technique of preoperative embolization and the application of early inflow control, adoption of the anterior approach and anatomical resection enabled us to curtail intraoperative hemorrhage and mitigate the need for transfusion.

To conclude, laparoscopic resection of extremely GH can be safely accomplished by experienced hepatobiliary surgeons with proper preoperative and intraoperative planning, and appropriate use of modern gadgets. The laparoscopic anterior approach is a safe and effective method to restrict blood loss during minimally invasive resection of GH.
